# A Human Motion Data Capture Study The University of Liverpool Rehabilitation Exercise Dataset

**DOI:** 10.1038/s41597-025-05099-1

**Published:** 2025-05-08

**Authors:** Nikhil Reji, Kristiaan D’Août, Sebastiano Fichera, Paolo Paoletti

**Affiliations:** 1 School of Engineering, University of Liverpool, L69 3GH Liverpool, UK; 2 Institute of Life Course and Medical Sciences, University of Liverpool, L7 8TX Liverpool, UK

**Keywords:** Rehabilitation, Rehabilitation, Computer science

## Abstract

The increasing accessibility of motion tracking technologies has resulted in a large amount of research focused on delivering exercise-based interventions remotely, coined under the term telerehabilitation. High quality human motion data is an essential component in the development and evaluation of Human Action Recognition (HAR) research, which plays a large role in exercise-based telerehabilitation. However, there is a lack of such human motion datasets for this domain, which hinders fast progress. This work presents a new human motion dataset named University of Liverpool Rehabilitation Exercise Dataset (UL-RED) containing 22 non-specialised exercises across 10 subjects and three data modalities: marker-based and marker-less motion tracking, and depth data. This dataset is the first to include motion repetitions of varying motion speeds, where subjects performed repetitions at a normal, fast, and slow pace. A total of 1,320 recordings were collected across the three data modalities, with over three hours of marker-based and marker-less motion tracking. This dataset is not only useful in the telerehabilitation landscape, but also within the wider field of HAR.

## Background & Summary

An artificial systems ability to understand human movement and categorise them is defined as Human Action Recognition (HAR), and it is commonly comprised of two stages: pose estimation and action recognition. Popular human motion datasets within the computer vision research landscape were designed to capture a broad range of individual and between-individual actions for the purpose of training and evaluating HAR algorithms. However, these datasets were created with no focus on an application space, which is reflected in the wide range of motion categories they contain. The Carnegie Mellon University Motion Capture database (CMU MoCap)^1^ was one of the earliest datasets containing motions categorised by locomotion, physical activities and sports, between-individual interactions, and scenario-based motions. These were collected using the Vicon infrared motion capture system (Vicon, USA) with 41 retro-reflective markers and stored using the Acclaim Skeleton File (ASF)/Acclaim Motion File (AMC). Such marker-based systems are commonly used within the biomechanics community due to the precise and reliable tracking capabilities^2^. The advent of marker-less tracking and implementation in telerehabilitation popularised by the Microsoft Kinect^3^, have provided inexpensive alternatives to marker-based motion capture, although at the cost of tracking precision^4,5^. The Microsoft Research Action 3D (MSR Action 3D)^6^, Joint-annotated Human Motion Data Base (JHMBD)^7^, and the Nanyang Technological University RGB + D (NTU RGB + D)^8^ are some of the other commonly used human motion datasets within general HAR research obtained using only marker-less motion tracking.

However, these datasets were created for general HAR research, which results in a wide range of motion categories: from sports related movements to daily actions such as eating and drinking. This has limited their use in exercise-based telerehabilitation research, as only a small subset of these motion categories match exercises seen in this domain. This absence of high-quality human motions in the rehabilitation space is evident by the lack of such datasets in the public domain. To the author’s best knowledge there are only six publicly available human motion datasets that focus purely on rehabilitation exercises that capture at a minimum marker-less human motion data. These are listed in Table 1 alongside the collected University of Liverpool Rehabilitation Exercise Dataset (UL-RED) dataset.

**Table 1:** A comparison between publicly available rehabilitation based human motion datasets and the presented UL-RED dataset.

Name and Year of Publication	Capture Method(s)	Labelled Repetitions	Motion Categories	Motions	Subjects
K3Da^19^ 2015	Marker-Less (Kinect)	No	Short Physical Performance Battery, Timed Up-And-Go, and Balance	13	54
UI-PRMD^12^ 2018	Marker (Vicon), Marker-Less (Kinect)	Yes	Standing rehabilitation exercises	10	10
KIMORE^13^ 2019	Marker-Less (Kinect V2)	No	Standing exercises for lower back pain	5	78
IRDS^20^ 2021	Marker-Less (Kinect)	Yes	Seated, standing, wheelchair, frame support arm, arm and leg range of motion exercises	9	29
Keraal^14^ 2022	Marker-Less (Kinect V2) & Marker (Vicon)	No	Upper body rehabilitation exercises	3	21
mRI^15^ 2022	Marker-Less (mmWave, RGB & Depth from Kinect V2, and IMU data)	No	Standing rehabilitation exercises	12	20
UL-RED^16^ 2024	Marker (OptiTrack), Marker-Less (Orbbec Persee + Nuitrack)	Yes	Seated, standing, and time variant rehabilitation exercises	22	10

The labelled repetitions column defines if a dataset contains motion recordings that are of a single repetition or have each repetition labelled withing a single recording.

The Kinect 3D active (K3Da) dataset collected exercises found in the Short Physical Performance Battery (SPPB)^9^ test, Time Up-And-Go (TUG)^10^ test, and balance. This is one of the first human motion datasets based on rehabilitation exercises captured using marker-less motion tracking, more specifically Microsoft Kinect^11^. Each subject was given three attempts to record a motion, but it was not clear if repeated attempts held correct movements, which left some ambiguity to parts of the dataset. Repetitions, for example the chair rise exercise which had five repetitions, were captured in a single recording without any ground truth labels. This makes it challenging to validate if a HAR algorithm correctly identified all repetitions. Moreover, although the motions were chosen from clinical rehabilitation assessments, they do not cover seated exercises which are also promoted in exercise-based rehabilitation. The exclusion of seated movements is also seen in the University of Idaho – Physical Rehabilitation Movements Data Set (UI-PRMD)^12^, the Kinematic Assessment of Movement and Clinical Scores for Remote Monitoring of Physical Rehabilitation (KIMORE)^13^ dataset, Keraal^14^, and mRI^15^.

UI-PRMD contains both marker-based and marker-less motion data, captured using the Vicon optical tracking system and the Microsoft Kinect^11^, respectively. Ten repetitions of ten exercises were recorded for each subject, with each repetition segmented to individual files. The dataset also contained incorrectly performed movements, for example performing a squat with an incorrect posture. However, subjects were not instructed to create specific sub-optimal movements and, without any measurement of this, it cannot be quantified to what extend the movement was incorrect. These incorrect movements could only be used to validate if a HAR algorithm could correctly identify an exercise even if it was performed sub-optimally. Furthermore, a large limitation of this dataset is the inconsistency of dominant side exercises across subjects, and within each exercise. For example, seven subjects performed the inline lunge with their left side and three with their right side.

IntelliRehabDataSet (IRDS) is similar to the K3Da dataset and only contains marker-less motion data obtained using the Microsoft Kinect^11^. Nine exercises across 29 subjects, 15 of which were patients and 14 part of a control group of healthy individuals, were collected targeting seven arm and two leg movements. An average age of 43 years was seen for the patient group, and 26 years for the control group. The patient group consisted of individuals with a diverse range of health conditions: five with spinal cord injuries, five that have had stroke, one that had a brain injury, one with a neurological condition, one that had an arm injury, one with a fractured femur, and one that had a prosthetic leg up to the knee. Subjects performed exercises in a wide range of situations: standing, seated, wheelchair, and frame support. Motions are segmented into individual files representing each motion and a correctness label indicating if the subject performed the motion correctly (1) or incorrectly (0). However, the number of correct and incorrect motions varied across subjects and motion categories, causing a large imbalance. Across movement situations, the imbalance saw 1215 movements performed standing, 952 seated, 359 on a wheelchair, and 51 using a frame support.

The KIMORE dataset captures five exercises focused on lower back pain across 44 healthy subjects and 34 subjects with motor dysfunctions. It is set apart from the rest of the datasets by the addition of clinical performance scores from an expert’s assessment of each subject recording. The selected exercises, chosen by the authors due to the low to no body occlusions, are limited to lower back pain exercises. For general telerehabilitation this dataset does not represent the wide variety of movements that are commonly seen in exercise-based rehabilitation. Similarly, the Keraal dataset also focused on lower back pain rehabilitation exercises. The authors captured three upper body exercises across 21 patients, 12 with lower back pain and 9 healthy. Marker-less data was captured across all subjects using the Microsoft Kinect, while marker-based data was captured for only three healthy subjects. In addition, the RGB data was used to compute 2D skeletal data using the OpenPose algorithm. Correct and incorrect movements were recorded and labelled, with time labels of where errors occurred, by two medical professionals in physiotherapy.

The mRI Dataset captured 12 standing rehabilitation exercises across 20 healthy subjects. Depth, RGB, amd mmWave data modalities were used to compute marker-less motion data for each exercise, where depth and RGB were captured using the Microsoft Kinect. Only a single repetition was recorded per subject, and it is not indicated if these repetitions were correctly performed or not. This dataset is unique for capturing human motion using mmWave radar sensors that only produces 3D point cloud data. The ability to record human motion using anonymous data capture methods such as mmWave is highly beneficial in rehabilitation applications, and in general healthcare applications, where patient data privacy is of importance.

The lack of seated and standing motions across a variety of movement categories, lack of marker-based ground truth alongside marker-less motion data, and the movement inconsistencies across subjects were the main motivation behind creating the UL-RED dataset.

This work presents a new dataset containing 22 seated and standing rehabilitation exercises across three data modalities and 10 healthy adult subjects. Five male and five female subjects between the ages of 18 and 65 were recruited. Male subjects had an average height of 1.80 ± 0.12 (mean ± standard deviation) m with an average arm span of 1.53 ± 0.12 m. Female subjects have a lower average height of 1.67 ± 0.03 m with also a lower average arm span of 1.40 ± 0.05 m. For each exercise, two recordings were captured, the first of a single repetition, and the second of a three time varied repetitions. The latter recording captured subject’s performing the motion at a normal, fast, and slow pace, in that order at their own intuition. A total of 1,320 recordings were collected using marker-based and marker-less motion tracking, alongside depth data.

### Summary

**Fig. 1:**
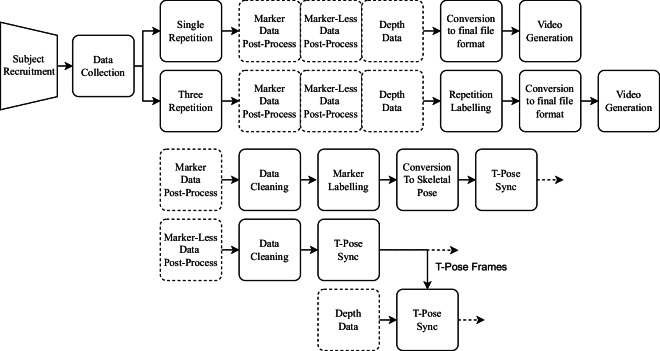
The UL-RED marker, marker-less, and depth subject data collection workflow. Single repetition data collection simply requires data post-processing and conversion to the final file format. Three repetition data collection required an additional manual repetition labelling stage after post processing. Marker-based data post-processing consisted of automated and manual marker data cleaning, labelling, and conversion to their final skeletal pose layout. For example, calculating the midpoint between two markers to create a joint representing the centre of the wrist. Marker-less data post processing only involved cleaning data errors. All post-processing methods also performed a final T-Pose synchronisation that used software based methods to align each motion recording. For each recording, the three data modalities were combined into a visual video format for easier access and review.

Figure 1 presents the data collection workflow that will be discussed in further detail in the Methods sections. The workflow shows the data collection process, from subject recruitment to post-processing the motion data, to obtain the finalised UL-RED dataset. Marker post-processing involves cleaning data samples, ensuring markers are labelled correctly, converting this marker set into a skeletal pose containing a collection of joint positions, and finally performing a synchronisation step using the T-Pose which subjects were instructed to perform at the start and end of every recording. Marker-less post-processing simply involves cleaning the data samples and performing the previously mentioned T-Pose synchronisation method in an effort to synchronise movements across data modalities. This is performed at the same time for the collected depth data which was used for the marker-less motion tracking computation. Each repetition within the three repetition recording is manually labelled to form segmented repetitions. Finally, the marker-based and marker-less data are converted to the Acclaim Motion Capture (AMC) and Acclaim Skeleton File (ASF) file format, whereas the depth data is stored as 16 bit binary depth files. For each motion recording, these three synced data modalities are rendered to a video format allowing for straightforward viewing of the dataset.

## Methods

Over three hours of marker-based and marker-less motion was captured with ethical approval from the University of Liverpool research ethics committee (Reference #10259). Participants provided full consent, via a signed consent form, in sharing and using their anonymised data through the University of Liverpool Data Catalogue to support other future research.

### Subject recruitment

Healthy adults are defined as adults who can complete the full range of motions for all exercises within the UL-RED dataset, with no musculoskeletal problems that limit this. The main exclusion criteria are that subjects did not have any injuries that affected normal biomechanics within the last six months and they did not have any dangerous medical conditions or any conditions potentially limiting biomechanics such as asthma, chronic injury, or pregnancy.

Table 2 shows the height, arm span, and sex of each subject. Male subjects had an average height of 1.80 ± 0.12 m with an average arm span of 1.53 ± 0.12 m. Female subjects have a lower average height of 1.67 ± 0.03 m with also a lower average arm span of 1.40 ± 0.05 m. All subjects had an average height of 1.73 m and an average arm span of 1.47 m. Height measurements were collected from the y-axis positional data of the head marker, which is located at the highest point on the subject’s skull. Arm span was calculated by computing the Euclidean distance between the left and right palm marker 3D position. These two measurements were taken while the subjects were in the T-Pose.

**Table 2:** Subject’s height, arm span, and sex.

Subject	Height (m)	Arm Span (m)	Sex
S01	1.66	1.38	F
S02	1.65	1.40	F
S03	1.65	1.37	F
S04	1.99	1.69	M
S05	1.72	1.50	F
S06	1.83	1.63	M
S07	1.64	1.37	M
S08	1.66	1.34	F
S09	1.72	1.44	M
S10	1.82	1.53	M

Body measurements were obtained from marker motion data during the T-Pose. F = Female. M = Male.

All subjects were instructed on specific clothing requirements prior to their allocated session to ensure optimum marker-based and marker-less tracking. This included avoiding dark coloured and loose fitting clothes, wearing collarless and sleeveless t-shirts, wearing low cut shoes and socks, and long hair had to be tied up using hair bobbles provided on the day. To allow for head marker placement, each subject was provided with a black motion cap. The participant information sheet describing all aspects of the data collection study, visual motion descriptions of the exercises the subjects have to perform, and the consent form that will be signed on the day were sent one week prior to data collection for subjects to get familiarised with.

### Exercise selection

For this data collection, non-specialised rehabilitation exercises were selected. These exercises are low risk movements that can be performed by patients safely in the comfort of their homes. This selection of upper body, lower body, and whole body exercises are commonly seen in the rehabilitation space, especially for older individuals. 22 exercises were selected based on publicly available exercise guidance by the National Health Service (NHS) in the United Kingdom (UK) (www.nhs.uk/live-well/exercise/physical-activity-guidelines-older-adults), and using insight from shadowing sessions at a local frailty therapy ward. This selection consisted of 6 seated and 16 standing movements, performed across all three body planes of movement: coronal, sagittal, and transverse. Table 3 defines the 22 exercises along with a short description of the movement. For each exercise recording, subjects were instructed to perform a T-Pose at the start and end of each recording. For asymmetric repetitions, such as the knee raise, subjects were instructed to perform with the left side first and then the right side. All exercises describe static movements, apart from FullFrontalsidewaysStep, where the subject’s whole body does not leave the original position in the room. For each motion, a single repetition and three-time varied repetitions were collected. The latter collected subjects performing the motion at a normal, fast, and slow pace, in that order. Subjects were guided to perform these three speeds by their own intuition.

**Table 3:** The 22 exercises captured in the UL-RED dataset.

Motion Name	Description
SitToStand	From standing transition to seated position and return
MiniSquat	Raise arms forwards in full extension with knees bent at an angle of 45 degrees
CalfRaise	While placing the ball of the foot on the ground, raise heel
SidewaysLegLift	Sweep left leg sideways in full extension, repeat for right leg
LegExtensionBackward	Sweep left leg up and backwards in full extension, repeat for right leg
LegExtensionForward	Sweep left leg up and forwards in full extension, repeat for right leg
BicepCurls	Bend elbows upwards and towards the chest, and return
SidewaysBend	Slide left hand down to the left knee while keeping arm in full extension, repeat for right side
SidewaysStep	Move left leg one step to the left, return and repeat for the right leg
KneeRaise	Raise left knee to form a 90 degree angle, repeat for right knee
ArmRaise	Raise both arms sideways in full extension until they meet above the head, and return
ForwardArmRaise	Raise both arms forwards in full extension until they are parallel to the floor, and return
FullFrontalSidewaysStep	Move the body one step to the left, return and repeat to the right
KneeRaiseWithOpHandTouch	Raise left knee to form a 90 degree angle and simultaneously bring right hand to left knee, repeat for right knee and left hand
HipHoops	Bring left hip outwards to the left and follow a single revolution circular pattern anti-clockwise and return
FrontalChestPress	Raise both arms sideways in full extension until they are parallel to the floor and sweep arms inwards till they meet, and return
SeatedLegStretch	Extend left leg forwards until full extension, repeat for right leg
SeatedChestStretch	This is the FrontalChestPress in the seated position
SeatedUpperBodyTwist	Place arms on chest in a X position and twist upper body to the left, repeat for the right
SeatedHipMarch	This is the KneeRaise in the seated position
SeatedArmRaise	This is the ArmRaise in the seated position
SeatedNeckRotation	While keeping upper body facing frontally, turn neck to the left, repeat for the right

Seated movements have the “Seated” prefix to their names. The motion names follow the same naming convention used in the data records.

### Marker motion capture

**Fig. 2:**
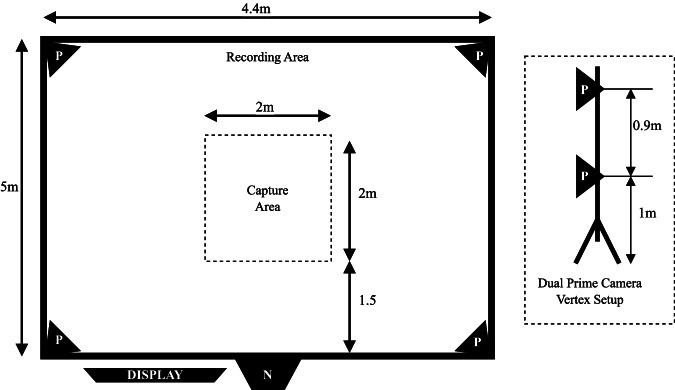
OptiTrack infrared camera (denoted by P) setup, recording area and capture area dimensions, and positions of the marker-less sensor (denoted by N). At each corner of the recording area, denoted by P, two Optitrack 17 W Prime cameras were setup facing the capture area. The vertical Optitrack camera setup ensured every marker was captured by at least three cameras to enable 3D reconstruction using the Optitrack Motive software. The marker-less sensor, Orbbec Persee, is placed at the midpoint of the longest side of the recording area, facing directly towards the capture area. The marker-less tracking output was then displayed in real-time using a digital display that participants can use to aid in their data capture. The capture area was significantly smaller than the recording area due to the space constraints of the marker-less tracking system.

Marker data was captured using an OptiTrack (NaturalPoint Inc., Corvallis, USA) system with eight infrared OptiTrack 17 W Prime cameras placed at each vertex of a 4.4 by 5 meter recording area, capturing marker data at a sample rate of 250 Frames Per Second (FPS). At each vertex, two Prime cameras are placed at 1 meter and 1.9 m from the floor to provide a wide field of view. Camera view angles were manually adjusted to ensure the lower cameras captured the lower body movements and upper cameras capture all upper body movements. To ensure this capture volume, and to align with the limited capture volume of the marker-less motion tracking system, a 2 by 2 meter capture area was setup at the centre of the recording area. Figure 2 shows the marker-based and marker-less sensor setup alongside the capture area location. To aid subjects in performing the exercises, a digital display was placed in the subjects’ view projecting the real-time marker-less motion tracking data. This not only helped ensure optimal motion tracking by both the subject and data collector, but also as a visual aid in ensuring correct exercise form.

**Fig. 3:**
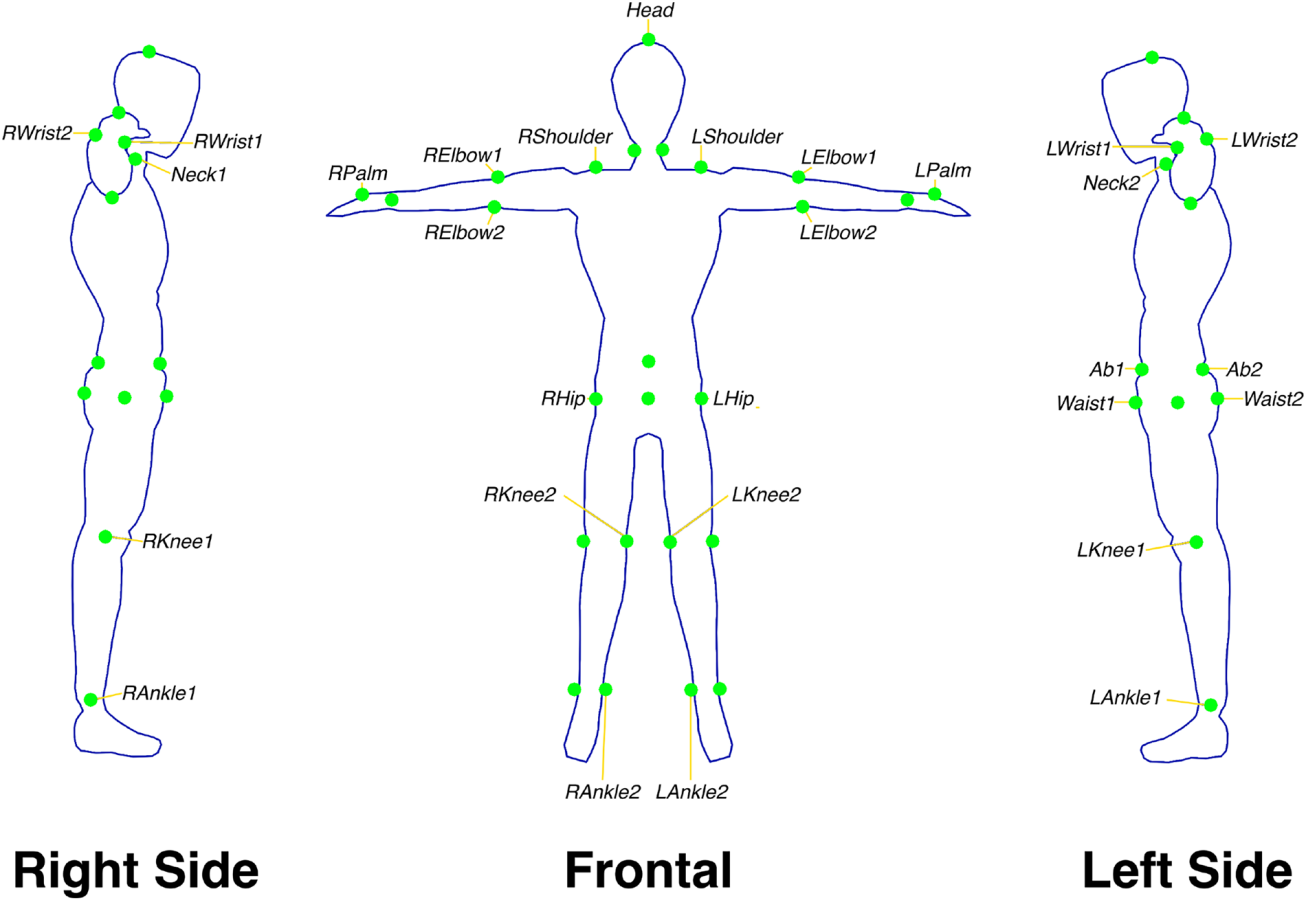
The marker placement of all 29 markers used to capture marker motion data with the OptiTrack system. Markers were placed by the data collector using skeletal and visual landmarks to allow for the capture of joint centres at a later stage. These markers were selected to represent skeletal pose layouts similar to ones seen from marker-less systems. All markers were attached either directly to the skin using skin friendly adhesives or on elastic cloth wrapping for areas such as the left hip joint. The head joint marker was attached onto a motion capture cap the participants wore.

To capture the subject’s movements, 29 retro—reflective markers were attached either directly on their skin using skin friendly adhesives or over clothed areas using tightly wrapped elastic cloth, following the marker layout in Fig. 3. To ensure consistency of marker placements between subjects, great care was taken to place markers using skeletal reference points where possible. For markers that do not directly reference such points, such as the waist marker, visual references were used to determine the correct placement, e.g. using the position of the left and right hip markers. The reference points used for all 29 markers are shown in Table 4. This marker layout was chosen to represent a similar but unique skeletal definition to existing marker-less motion tracking systems.

**Table 4:** Reference points used for positioning of 29 retro-reflective markers on the participants.

Marker Label	Skeletal/Visual Reference
Head	Highest visual point on the skull while standing upright
Neck1	Right side of the vertical midpoint of the neck
Neck2	Left side of the vertical midpoint of the neck
LShoulder	Top of the left clavicle end towards the left upper arm
LElbow1	Left outer point along the left elbow rotation axis during flexion/extension
LElbow2	Right outer point along the left elbow rotation axis during flexion/extension
LWrist1	Left outer point along the carpal bones rotation axis during left wrist flexion/extension
LWrist2	Right outer point along the carpal bones rotation axis during left wrist flexion/extension
LPalm	Centre point of metacarpal bones on the back of the left hand
RShoulder	Top of the right clavicle end towards the right upper arm
RElbow1	Right outer point along the right elbow rotation axis during left elbow flexion/extension
RElbow2	Left outer point along the right elbow rotation axis during left elbow flexion/extension
RWrist1	Right outer point along the carpal bones rotation axis during right wrist flexion/extension
RWrist2	Left outer point along the carpal bones rotation axis during right wrist flexion/extension
RPalm	Centre point of metacarpal bones on the back of the right hand
Ab1	Outer point located at the midpoint between the base of the ribs and Waist1 marker on the frontal body side
Ab2	Outer point located at the midpoint between the base of the ribs and Waist2 marker on the rear body side
Waist1	Frontal midpoint between the LHip and RHip marker
Waist2	Rear midpoint between the LHip and RHip marker
LHip	Left outer point along the rotation axis during left hip flexion/extension
LKnee1	Left outer point along kneecap rotation axis during left knee flexion/extension.
LKnee2	Right outer point along kneecap rotation axis during right knee flexion/extension.
LAnkle1	Left outer point along left talus bone (ankle) rotation axis during left foot flexion/extension
LAnkle2	Right outer point along left talus bone (angle) rotation axis during left foot flexion/extension
RHip	Right outer point along the rotation axis during right hip flexion/extension
RKnee1	Right outer point along kneecap rotation axis during right knee flexion/extension
RKnee2	Left outer point along kneecap rotation axis during right knee flexion/extension
RAnkle1	Right outer point right talus bone (ankle) rotation axis during right foot flexion/extension
Rankle2	Left outer point right talus bone (ankle) rotation axis during right foot flexion/extension

Before each subject’s recording session, a two-stage camera calibration is performed following the OptiTrack guidelines. This consisted of a 3D position and orientation calibration using the passive OptiTrack CW-500 wand, and a ground plane calibration using the OptiTrack CS-200 calibration square. All calibration and data capture were performed using the accompanying software Motive version 2.1.1.The coordinate systems origin point is at the centre of the capture area, on the floor, with the positive z axis facing away from the marker-less camera (refer to Fig. 2), positive y axis directed upwards from the origin point, and positive x axis directed towards the left of the marker-less camera (when facing in the direction of the positive z axis).

### Marker data post-processing

Once the marker data has been collected, it is first labelled and cleaned following the marker labels in Fig. 3. The first visible frame is selected and each marker is labelled manually. With this reference frame, the Motive software then attempts to automatically label markers across all frames. Commonly, there are gaps in tracking data from occlusions or mislabelling from the automated process. Unlabelled markers are corrected by manually traversing the frames and labelling data where possible. Missing marker data, under 500 successive frames, are filled using the Motive linear interpolated gap filler tool. For marker data that exceed this gap, the Motive pattern based interpolator is used which interpolates a marker position based on two neighbouring markers. This assumes that the neighbouring points form a rigid body, which can be made for skeletal data. For example, the LElbow1 marker data can be interpolated using this approach by referencing both LElbow2 and LWrist1 markers. Once all data is labelled and cleaned, they are exported as a Comma Separated Value (CSV) file.

**Fig. 4:**
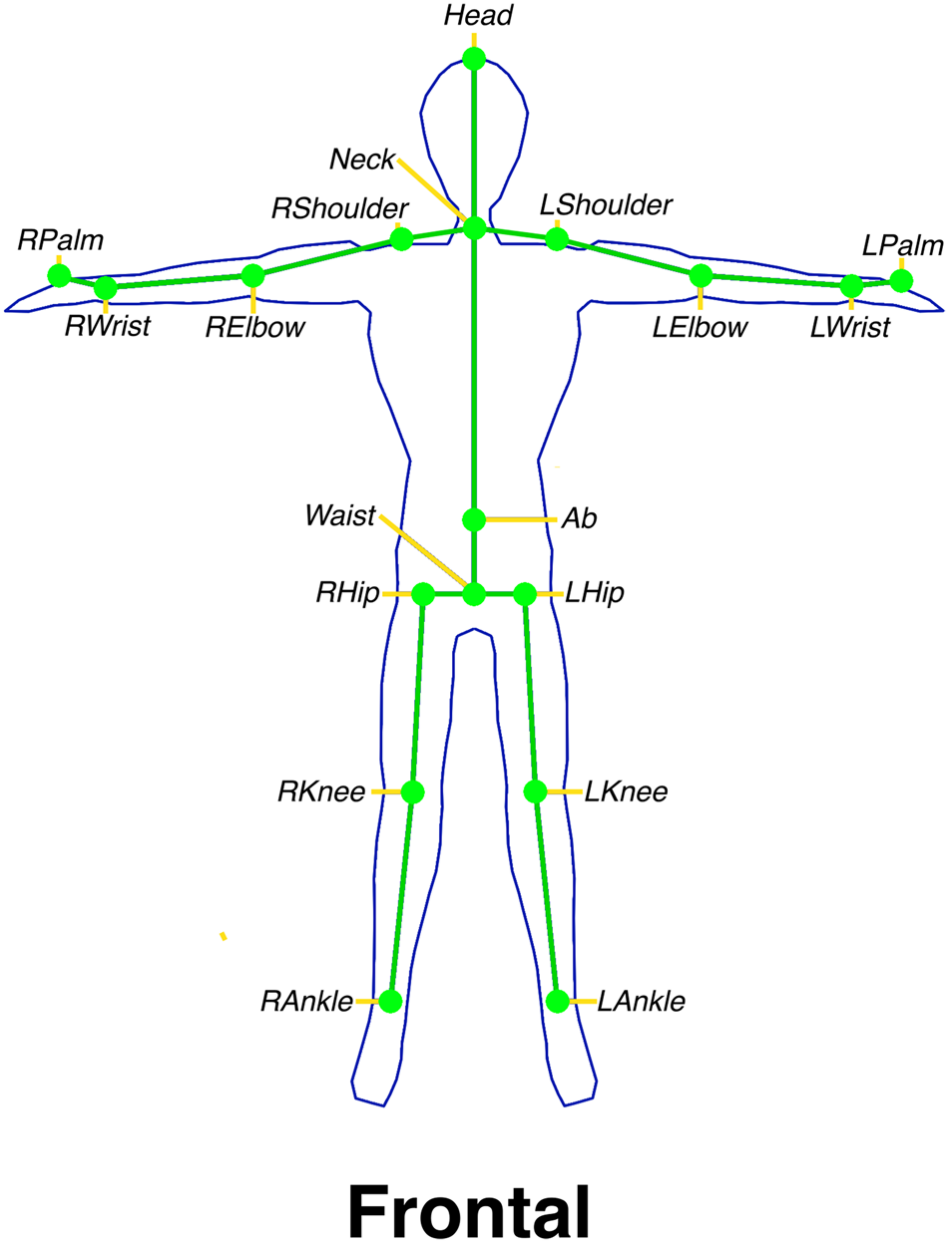
The resulting 18 joint skeletal definition after transforming the marker data. The marker-based data were used to calculate the joint centers that replicated the 3D positions outputted by marker-less tracking systems. The head marker and palm markers performed a one-to-one mapping of its position for the head and palm joints. The hip joints were computed using the midpoint of the hip, and both waist markers (Waist1 and Waist2). All other joint positions were computed using the midpoint between two markers.

The raw marker data is then transformed into the pose definition shown in Fig. 4. For the head, left shoulder, right shoulder, left palm, and right palm markers, a direct transformation is performed. This copies the 3D marker position using the mapping

$$f:\left(x,y,z\right)\to (x,y,z)$$

For all other marker pairs, denoted by either 1 or 2 in its suffix, excluding left hip and right hip, a pairwise transformation is performed. The corresponding joint position in Fig. 4 is calculated from the midpoint between two 3D marker positions using the mapping

$$f:\left({v}_{1},{v}_{2}\right)\to \left(\left(\frac{{v}_{1}^{x}+{v}_{2}^{x}}{2}\right)+\left(\frac{{v}_{1}^{y}+{v}_{2}^{y}}{2}\right)+\left(\frac{{v}_{1}^{z}+{v}_{2}^{z}}{2}\right)\right)$$

Where *v*_*x*_ denotes a 3D position vector containing x, y, and z positional values. For the left and right hip joints, the midpoint between each hip marker and the Waist1 and Waist2 markers were calculated. This is simply an extension of the pairwise transformation

$$f:\left({v}_{1},{v}_{2},{v}_{3}\right)\to \left(\left(\frac{{v}_{1}^{x}+{v}_{2}^{x}+{v}_{3}^{x}}{3}\right)+\left(\frac{{v}_{1}^{y}+{v}_{2}^{y}+{v}_{3}^{y}}{3}\right)+\left(\frac{{v}_{1}^{z}+{v}_{2}^{z}+{v}_{3}^{z}}{3}\right)\right)$$

These three processes completes the transformation of the marker layout shown in Fig. 3 to the skeletal layout shown in Fig. 4.

**Fig. 5:**
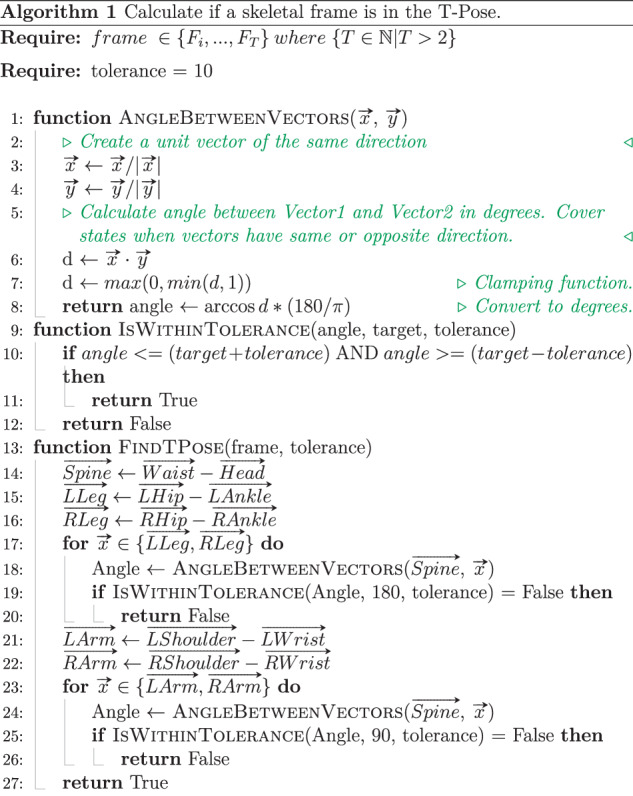
Pseudo code of the T-Pose cropping algorithm used to trim both marker and marker-less motion data. To find a T-Pose, the angle between the spine vector, defined by the vector between the waist and head joints, and both the left and right arms and legs vectors were used. The arm vectors were defined as the vector between the shoulder and wrist joints. The leg vectors were defined as the vector between the hip and ankle joints. A pose was considered to be in the T-Pose if the angle between the arms and spine were 90 degrees ± 10 degrees, and the angle between the legs and spine were 180 degrees ± 10 degrees.

Finally, a T-Pose crop is performed to ensure all recordings start and end with a T-Pose. Although great care was taken to ensure subjects always started and ended in a T-Pose, there were situations where a T-Pose was held for a long period of time. This stores unnecessary and duplicate motion data that does not contribute to the captured exercise. Hence, a walking T-Pose cropping algorithm is implemented which traverses the data forwards and in reverse to perform this crop. A T-Pose is defined by the angle between the spine vector, defined by the waist and head joints, and four vectors defined by the joint pairs: left shoulder and left wrist, right shoulder and right wrist, left hip and left ankle, right hip and right ankle. A T-Pose requires an angle of 90 degrees ± 10 degrees between each arm and spine vector, and 180 degrees ± 10 degrees between each leg and spine vector. Figure 5 presents the pseudo code of the implemented walking T-Pose cropping algorithm. The FindTPose function calculates if a skeletal frame is in the T-Pose using the previously defined angular constraints. This function is then iterated through frames until the last T-Pose is found. This walking method is performed forwards from the first frame with a step of one, and backwards from the last frame with a step of negative one. This method is also applied during the marker-less motion data post-processing which can aid in the synchronisation of the two motion data. The skeletal motion data is than stored in the Acclaim Motion Capture (AMC) file format which is detailed in the Data Records section.

### Marker-less & depth motion capture

**Fig. 6:**
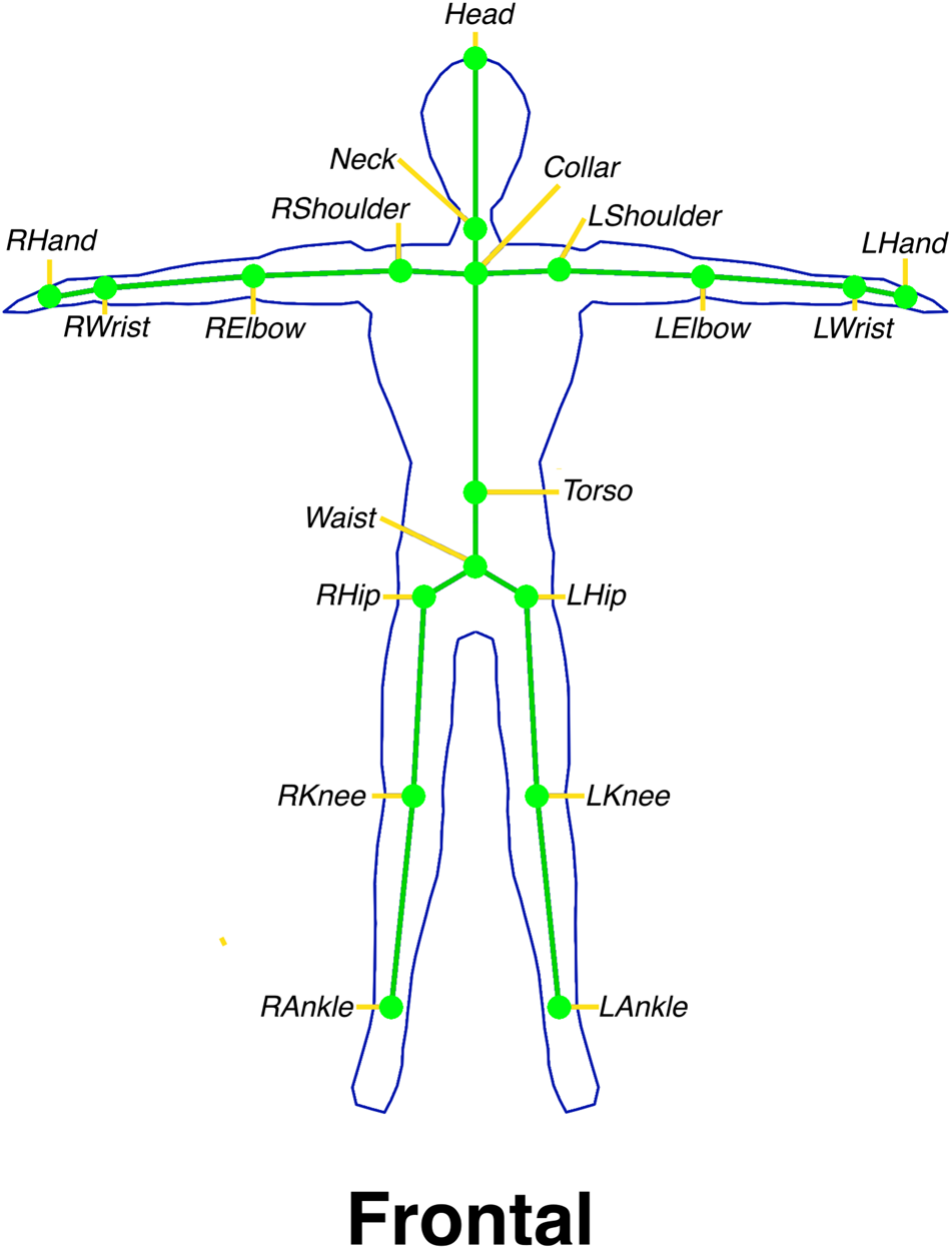
The Nuitrack marker-less 19 joint skeletal definition. The location of all joint 3D positions provided by the Nuitrack marker-less tracking system. This method of joint tracking uses infrared depth data that allows for real measurement of the depth of each joint position. This computation is performed in real-time and on the Orbbec Persee depth camera device used for the marker-less data collection.

Marker-less motion data was captured at a sample rate of 30 FPS using the Nuitrack 3D skeletal tracking library (Cvartel Inc., Covina, USA) that uses depth data to recognise skeletal poses in real time. The depth data was captured using the Orbbec Persee (Orbbec Inc., Shenzhen, China) depth camera computer version one. The Nuitrack library tracks a 19 joint skeletal pose shown in Fig. 6, with each joint defined by its 3D position relative to the depth sensors origin. Although the Nuitrack system can be paired with a wide variety of depth sensors, the Orbbec Persee version one was chosen due to its optimal depth tracking and onboard computing unit that can host the Nuitrack library. The depth data was collected simultaneously with the marker-less motion data, at a resolution of 160 by 120 pixels at 16 bit. Although all related datasets used the Microsoft Kinect for marker-less motion tracking (refer to Table 1) the decision to not use the Kinect was due to its deprecated hardware and software, which is a barrier to future use.

**Fig. 7:**
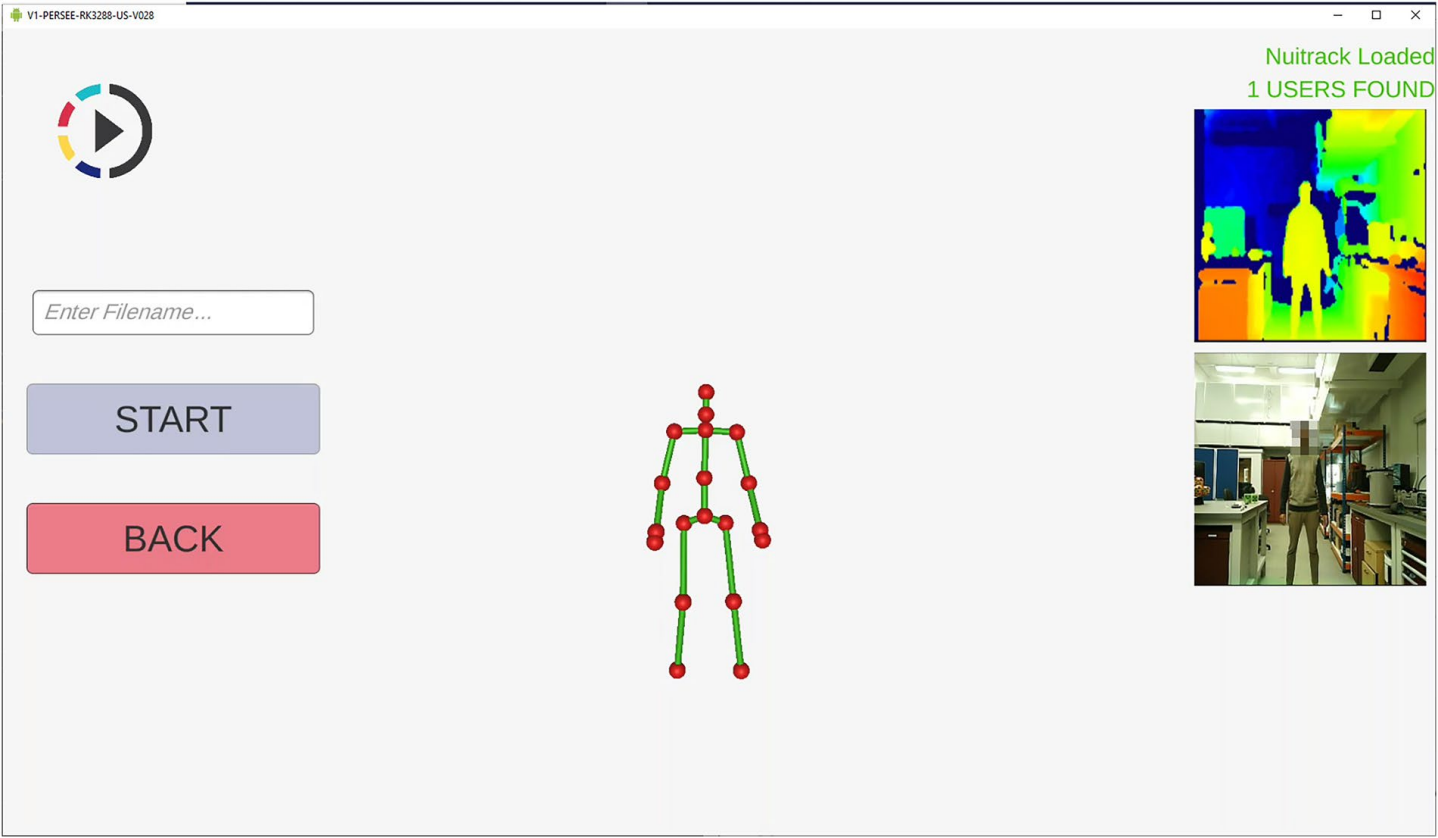
Nuitrack based marker-less motion tracking Android application running on the Orbbec Persee on board computing unit. This Android based application was developed to run on the Orbbec Persee platform to both aid in the capture of human motion data and provide visual aid to participants during data collection. This platform was interacted with remotely via an ethernet connection and the Android Debug Bridge (ADB) on the computing unit hosting the Optitrack Motive software. A unique filename was required to begin tracking, entered within the white text box. All files were saved locally on the device temporarily before transferring to their final secure research drive location.

To collect marker-less motion data and depth data using the Nuitrack library and the Orbbec Persee depth camera, a custom Android mobile application was developed using the Unity game engine which ran on the Orbbec Persee onboard computing unit. Figure 7 shows the recording screen of this application which was also displayed to the subjects using a digital display, as shown in Fig. 2.

The data collection implemented all the recommendations provided by the Nuitrack library for optimal marker-less motion tracking. The tracking area did not have direct sunlight exposure. The Orbbec Persee depth camera was placed at a height of 1.2 m from the floor. The capture area was between 1.5 and 3 m from the Orbbec Persee depth camera. The surrounding walls and ceilings of the capture area must have a gap of at least 40 cm. Finally, subjects were asked to avoid dark coloured clothing which can negatively impact motion tracking.

### Marker data post-processing

A three stage outlier filtering process is applied to reduce the effects of skeletal tracking errors where possible. In certain situations, the Nuitrack library would produce skeletal tracking joints with low confidence, which resulted in the joint position not being tracked. Such situations were recorded in the data collection stage as zero value 3D vectors. From visual inspection, there were tracking errors resulting in incorrect joint placements for certain intervals of time. The authors assume these errors are caused mainly by body occlusions but it is difficult to further evaluate this as the Nuitrack library code base is closed source.

The walking T-Pose cropping algorithm discussed previously, and defined in Fig. 5, was first applied to ensure every recording starts and ends in a T-Pose. This also helps aid in synchronisation between marker-based and marker-less motion data. Then, any joints that exceed their skeletal bone lengths in reference to their parent joint, excluding the waist joint, is labelled as not tracked using a zero value 3D vector. The bone lengths are referenced from the first T-Pose frame of each recording and outliers are found if their bone lengths are greater than a factor of 1.01 or less than a factor of 0.99. This 1% tolerance ensures consistent tracking across each frame, at 30 FPS each frame is captured every 33.33 milliseconds hence deviations greater that 1% can be an indication of inaccurate tracking.

These outliers, in addition to the untracked joints by Nuitrack, are filled using a forward fill approach in two passes. The first pass fills the outliers found by Nuitrack, then the second pass fills outliers found using the bone length method. The forward fill method takes the last valid tracked position of the joint and duplicates it across the consecutive outlier frames. This is performed in order of the joint hierarchy, starting with the root joint set to the waist joint. These steps attempts to fix most of the tracking errors contained in the marker-less data. However, it is not guaranteed to fix every tracking artefact produced.

### Data collection process

**Fig. 8:**
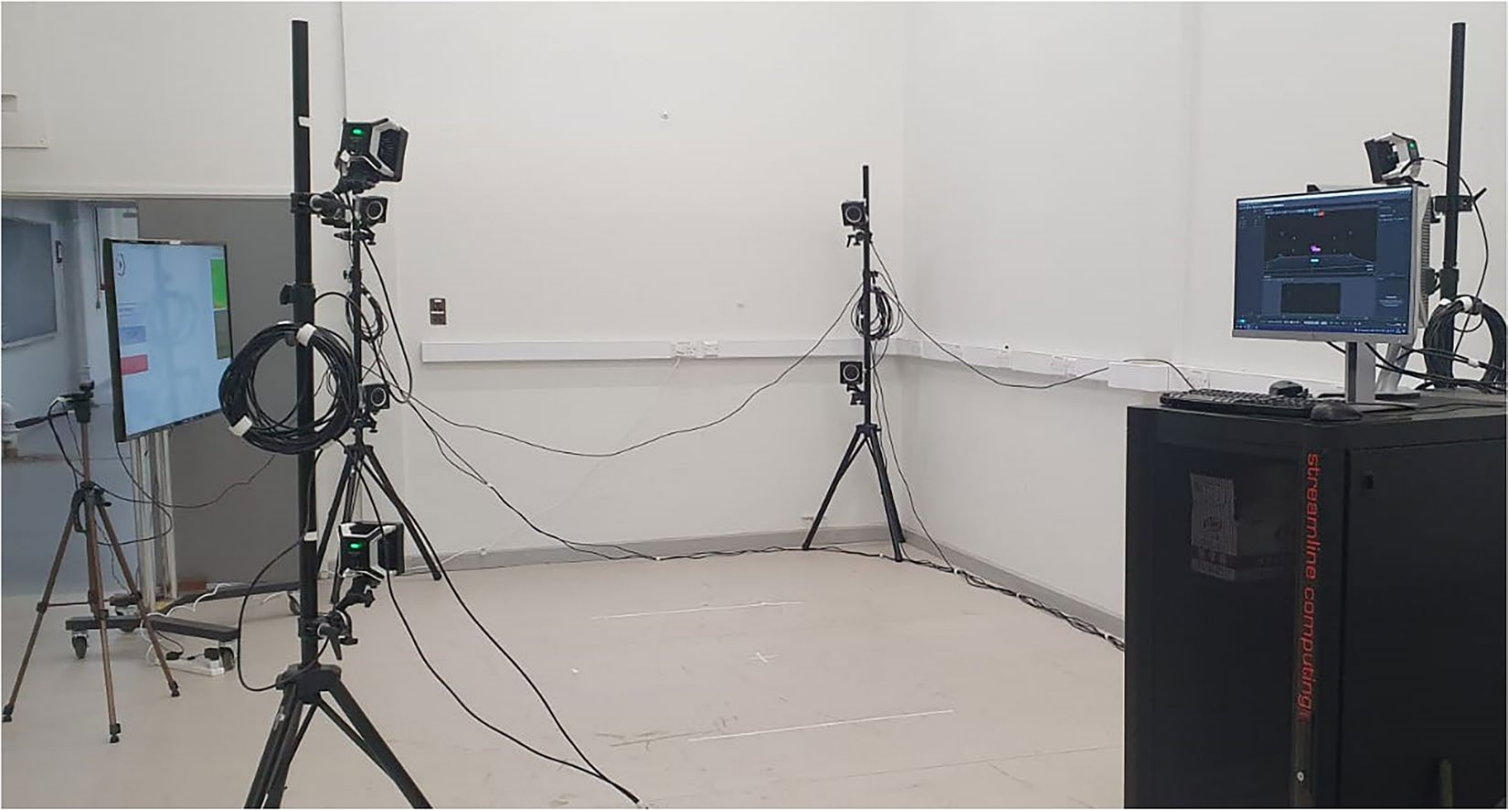
The OptiTrack (marker-based) and Nuitrack (marker-less) capture environment used in the UL-RED data collection study. This follows the same layout description in Fig. 2. All cables were routed to ensure low risk to participants during entry to and exit from the capture arena. The capture area was situated in a closed room with no direct sunlight to ensure minimal interference to both the marker-based and marker-less systems.

The marker-based and marker-less setup was presented in Fig. 2, with subjects performing exercises within the specified capture area. Figure 8 shows the real setup following the layout shown in Fig. 2. However, the OptiTrack and Nuitrack systems tracked motion in two isolated computing environments with no direct hardware synchronisation capabilities. To help synchronise these the marker-based, marker-less, and depth data, subjects performed the T-Pose at the start and end of each recording, in addition to automating the start of the data capture using mouse movement macros. This was achieved using the Auto Mouse Clicker by MurGee (Daanav Softwares, Goa, India) software which performed preset on-screen mouse interactions with a minimum delay of approximately 1 millisecond. To allow for this automated start, the Nuitrack recording application, shown in Fig. 6, hosted on the Orbbec Persee computing unit was streamed to the desktop computer hosting the OptiTrack Motive software using a direct ethernet connection, with the Android Debug Bridge (ADB), and the scrcpy library (www.github.com/Genymobile/scrcpy). Although great effort was taken to ensure synchronisation using this software-based approach, it is not guaranteed that both data modalities are perfectly synchronised.

## Data Records

All the motion data, processed and raw, along with the python scripts used to generate the data can be accessed through the University of Liverpool data catalogue^16^.

Processed marker-based and marker-less motion data are stored in the Acclaim Motion Capture (AMC) and Acclaim Skeleton File (ASF)^17^ formats, which were chosen due to their human readability. The AMC file stores all the frames within a motion recording, where a frame consists of a collection of 3D joint positions. The ASF file describes the skeletal joint definition for a specific subject containing the sample rate, joint hierarchy, axis order, bone lengths, and the subject’s joint positions describing a T-Pose. A custom python package was developed to easily parse the AMC and ASF files, which can be found on the public python package repository pypi, named asfamc-parser^18^.

**Fig. 9:**
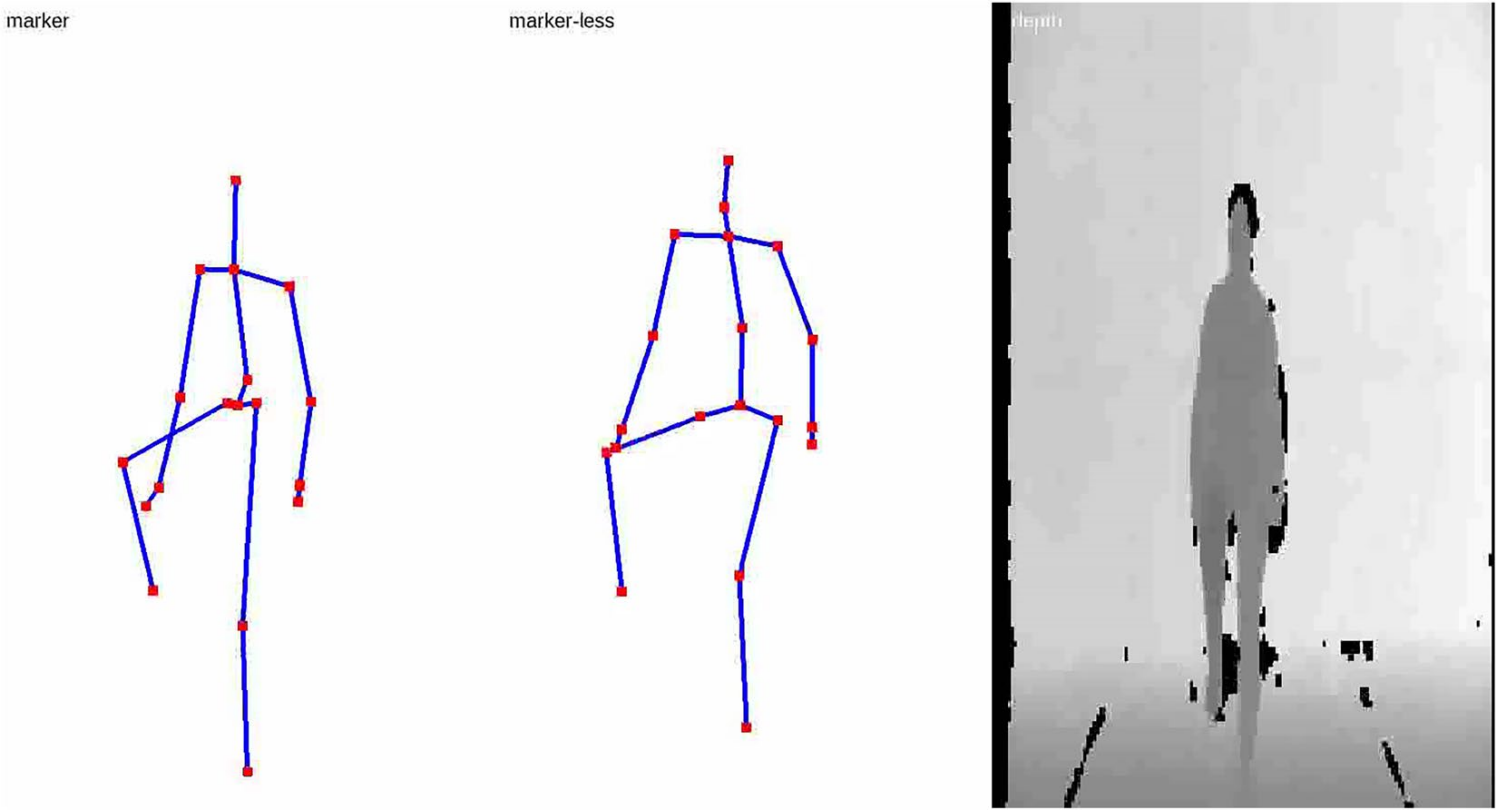
Marker-based, marker-less, and depth data video representation example. This visual video represents the three captured data modalities after all data post processing and synchronisation. The depth data is viewed using a frontal view, while the marker-based and marker-less data are positioned at the 45 degree angle to the view. Labels at the top of each recording indicate their source.

Raw marker-less data is also stored in the same ASF/AMC file format, while the raw marker position data, and all motion labels, is stored as a Comma Separated Value (CSV) file which was exported directly from the OptiTrack Motive software, used to capture marker data. Each recordings depth data is stored as a collection of binary files, each containing a single 16 bit depth frame at a resolution of 160 by 120 pixels. Lastly, the generated video representation of the motion data modalities are stored as an Audio Video Interleave (AVI) file, which can be easily viewed using the freely available VideoLAN software (VideoLAN, Paris, France). Figure 9 shows an example video frame.

### Naming convention

All motion data is stored using the following naming convention:

*{Motion Name}R{Number of Repetitions}S{Subject ID}*

Marker labels for the three repetition motions follow the naming convention 3Rep_S{SubjectID}, and is stored as a CSV file. Note, the marker-less three repetition labels were generated by transforming the marker labels using:

$${{label}}_{{marker}-{less}}={Ceil}\,\left(\left(\frac{{{label}}_{{marker}}}{{f}_{{marker}}}\right)\ast {f}_{{marker}-{less}}\right)$$

Where f_marker_ and f_marker-less_ refer to the sampling rates used: 250 FPS for marker and 30 FPS for marker-less. The Ceil function rounds the number to the next closest integer.

### File structure

**Fig. 10:**
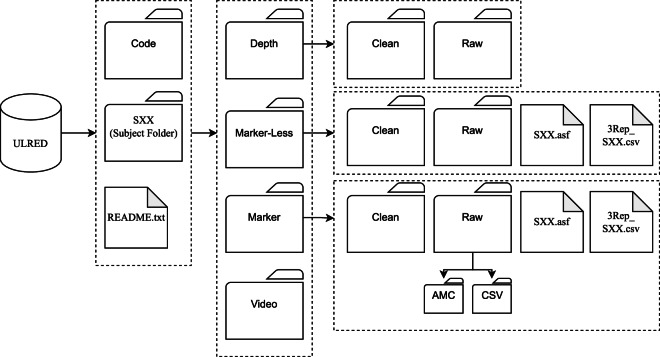
UL-RED folder structure. The folder structure and README files allow for an accessible data store that does not rely on external documentation. Data is organised by Subjects at a high level, data modality at middle level, and data processing stage at low level. For marker data, the CSV file contains the original unaltered exported data from the Optitrack motive software.

All motion data is structured at the top level by subject ID, then by motion data modality, and then by its condition (processed or raw), this is shown in Fig. 10. The README file contains all the information required to navigate these folders and instructions to run the python scripts contained in the code.

## Technical Validation

### Missing data

Although great effort was taken to ensure high quality recordings, there were four instances of data loss seen across all subject recordings: KneeRaiseWithOpHandTouchR3S10 and LegExtensionBackwardsR3S10 both have missing depth data, with the latter only missing depth data after the second repetition. SidewaysStepR3S10 has missing marker-less and depth data. Finally, SitToStandR3S06 has no end T-Pose. For three repetition motions there were several instances where subjects did not return to the base pose in between repetitions. For example, in the seated chest press subjects were guided to return to a resting seated position with their arms relaxed on the side after each repetition. There were 15 instances of this: MiniSquatR3S06, MiniSquatR3S07, MiniSquatR3S08, MiniSquatR3S09, SeatedChestStretchR3S04, SeatedChestStretchR3S05, SeatedChestStretchR3S06, SeatedChestStretchS08, SeatedUpperBodyTwistR3S03, SeatedUpperBodyTwistR3S04, SeatedUpperBodyTwistR3S05, SeatedUpperBodyTwistR3S06, SeatedUpperBodyTwistR3S07, SeatedUpperBodyTwistR3S08, SeatedUpperBodyTwistR3S09.

### Motion speed variances

**Fig. 11:**
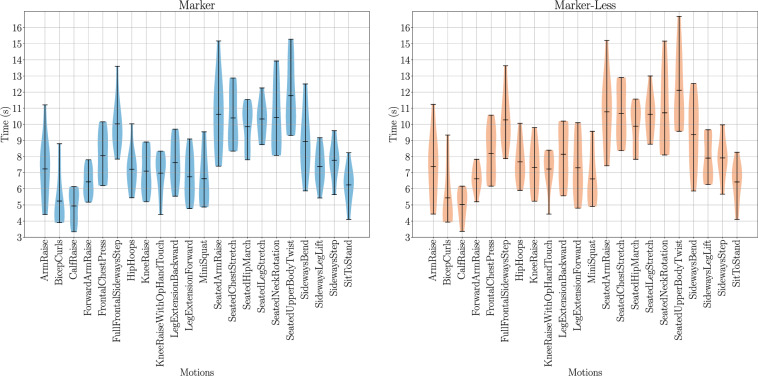
A violin plot of the durations for single repetition motions in the UL-RED dataset, the violin plot centerline represents the mean value for that distribution. For each plot the shaded area represents the distribution of samples with the upper and lower extremities denoted by the bar plot. Similar distributions of durations are seen across similar motions between marker and marker-less data. Seated motions, apart from sit to stand, are shown to take considerably longer than standing motions. This is a result of the extra transition period required from the standing T-Pose requirement. A wide variety of motion durations are recorded for each motion type, creating a diverse dataset that aim to capture real life variances in motions.

Single repetition motions are labelled as a normal paced movement, but this can vary between subjects, especially in the time domain. Although all subjects performed the motions as instructed, the duration taken to perform them can vary significantly. These variances for marker-based and marker-less data are presented as a violin plot in Fig. 11. The measured durations follow a similar trend for both marker-based and marker-less data which is evidence of good synchronisation. Seated motions are shown to take significantly longer to perform. This is a result of the T-Pose requirement at the start and end of each recording, as this adds a longer transition phase from the standing to seated position before beginning the movement. It is shown that a wide range of movement speeds are captured of subjects performing a normal paced movement.

**Fig. 12:**
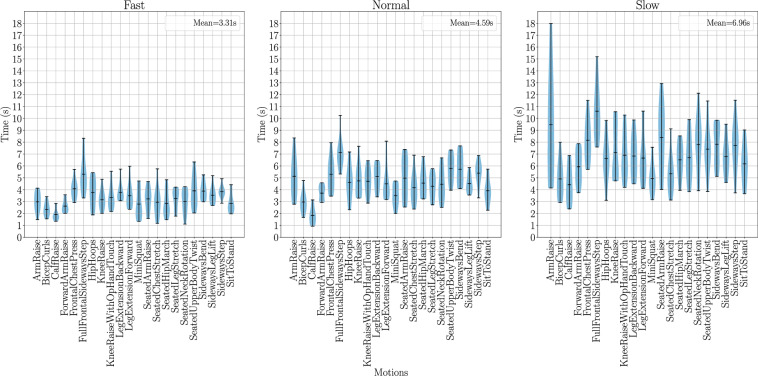
A violin plot of the durations for three repetition marker motion data in the UL-RED dataset, the violin plots centerline represents the mean value for that distribution. For each plot the shaded area represents the distribution of samples with the upper and lower extremities denoted by the bar plot. It is clearly seen that the duration of all motion types increase as the repetition speed decreases. However, there is still a wide variety of durations at each repetition stage due to natural human variances and also due to participants being guided to complete these repetition speeds with their own intuition. It is also shown that seated and standing motions do not have significant difference as seen in single repetitions. This is a result of there being no requirement to return to a T-Pose between repetitions.

This analysis was repeated for the three repetition motion data, shown as a violin plot in Fig. 12. This was performed only on marker-based motion data as the marker-less motion data three repetition labels were the transformation of the marker labels. The fast, normal, and slow motions show an increase in duration: fast movements performed with an average duration of 3.31 s, normal movements with 4.59 s, and slow movements with 6.96 s. The previously seen increase in duration among seated motions is not seen as there is no transition period between seating and standing poses in between repetitions. It is also interesting to see the large variation in slow movements across subjects, in comparison to the other repetition speeds. Each subject was instructed to perform these movements at their own intuition which can explain such variances.

### Angular evaluation

To evaluate the accuracy of the Nuitrack marker-less pose tracking, the marker-less motion data is compared against the marker-based motion data that is considered as the gold standard. The similarity between joint angles and bone length measurements were used to perform this comparison.

**Fig. 13:**
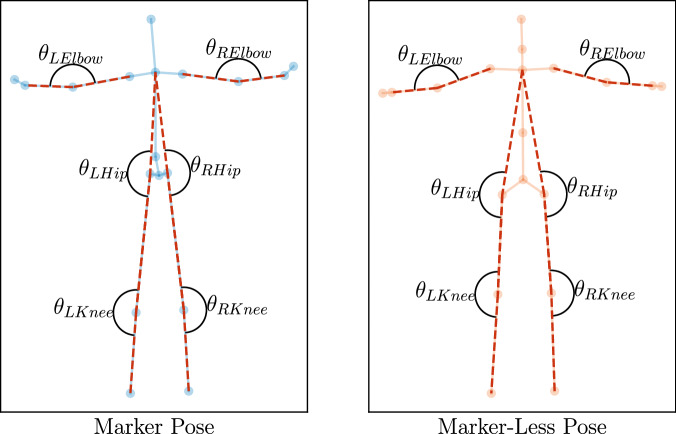
Marker-based and marker-less joint angles selected to compare marker-less pose tracking performance. Marker-based refers to motion data collected using the OptiTrack system, and marker-less refers to motion data collected using the Nuitrack system. These angles were selected to represent measurements that are similar between both pose layouts. For the elbow angle measurements, the vectors formed by the wrist, elbow, and shoulder joints are used. For the hip angle measurements, the vectors formed by the neck/collar, hip, and knee joints are used. For the knee angle measurements, the vectors formed by the hip, knee, and ankle joints are used.

Six joint angles that were similar between the two pose layouts were taken to perform a fair comparison. As shown in Fig. 13, this consists of the left knee, right knee, left hip, right hip, left elbow, and right elbow angles. These angles were selected due to their similar joint locations between both pose layouts.

The similarities between the marker-based and marker-less motion sequences were computed using the Mean Absolute Error (MAE) between all angular values across all frames in each recording, i.e.

$${MAE}=\frac{1}{n}\left(\mathop{\sum }\limits_{i=1}^{n}\left|{\theta }_{i}^{{marker}}-{\theta }_{i}^{{marker}-{less}}\right|\right)$$

**Fig. 14:**
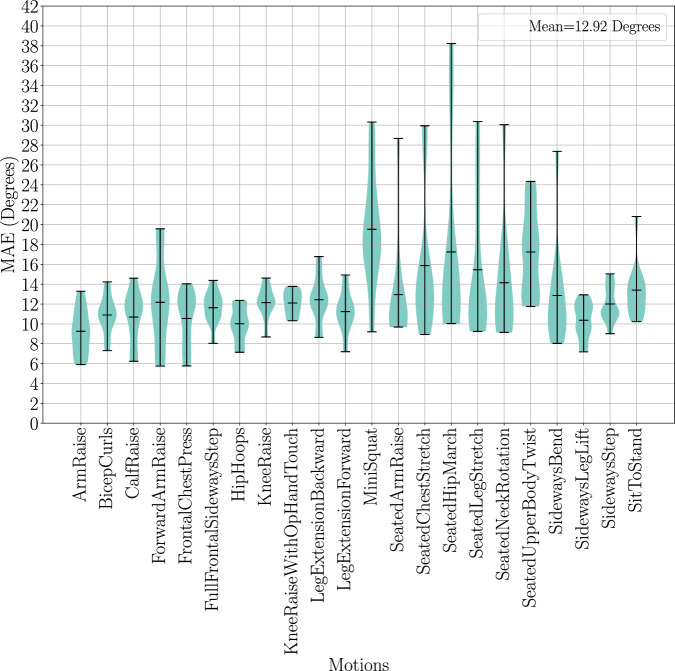
MAE comparison between marker-based and marker-less joint angles across all motions in the UL-RED dataset. The centerline in each distribution represents the mean value. The Mean Absolute Error computes the average of absolute difference between a joint angles marker-based and marker-less measurements, for all joint angles. Seated joint angles are shown to produce large outliers/extremes which can be attributed to inaccurate positioning of lower body joints, such as the hip joint, when in a seated position. This is due to the single perspective view of the depth sensor used for marker-less tracking that can degrade its performance during occluded scenarios, such as the hip location being occluded during a seated position.

Marker data is sampled at 250 FPS, while marker-less data was sampled at 30 FPS. To perform a fair comparison, the angle at every 0.5 s was compared. For marker data this was every 125 frames, and for marker-less data every 15 frames. Although synchronisation between these two data modalities was performed it cannot be guaranteed. Hence, certain motions may have more or less frames between marker and marker-less data. To alleviate this, the longest sequence between the two was truncated to the size of the shorter sequence to perform the MAE comparison. The violin plot in Fig. 14 shows the range of angular errors where an average MAE of 12.92 degrees was seen across all exercise motions.

Seated motions saw a large MAE of 15.48 ± 6.92 degrees (mean ± standard deviation), while standing motions had an average MAE of 11.95 ± 3.68 degrees. The median MAE values were 12.84 degrees for seated and 11.71 degrees for standing. This shows a positively skewed distribution for seated motions, where a large angular error can come from a few outliers. This can be indicative of large marker-less tracking errors due to body occlusions. Standing motions indicate a symmetrical distribution which describes all motions incurring a similar angular error. This larger angular difference in seated motions can be a result of the hip joints being occluded while seated, resulting in poor marker-less tracking performance.

### Bone-length evaluation

The marker-less joint location accuracy can be evaluated indirectly by comparing bone lengths: the length between two joint positions. A collection of eight bone lengths were computed:

- Left forearm: left elbow to left wrist
- Right forearm: right elbow to right wrist
- Left upper arm: left elbow to left shoulder
- Right upper arm: right elbow to right shoulder
- Left lower leg: left knee to left ankle
- Right lower leg: right knee to right ankle
- Left upper leg: left knee to left hip
- Right upper leg: right knee to right hip

Marker-less joint positions were converted to m (from millimetres) to match the marker units. For each exercise recording, only the first frame containing the T-Pose was used to compute the bone lengths. A bone length was computed by taking the Euclidean distance between the 3D position vectors *v*_*1*_ and *v*_*2*_ describing the two relevant joints, i.e.

$${bone\; length}=\sqrt{{({v}_{1}^{x}-{v}_{2}^{x})}^{2}+{({v}_{1}^{y}-{v}_{2}^{y})}^{2}+{({v}_{1}^{z}-{v}_{2}^{z})}^{2}}$$

For each bone length, the MAE is computed across all exercises and the mean, median, and standard deviation in millimetres (mm) was calculated and shown in Table 5.

**Table 5:** Mean Absolute Error between marker and marker-less bone lengths and their mean, median, and standard deviation (std) in millimeters.

Bone	Mean ± std (mm)	Median (mm)
Left Forearm	10.54 ± 6.35	9.46
Right Forearm	7.87 ± 9.60	4.8
Left Upper Arm	35.08 ± 20.77	32.12
Right Upper Arm	33.00 ± 13.32	31.36
Left Lower Leg	21.36 ± 13.68	19.06
Right Lower Leg	21.48 ± 14.67	12.89
Left Upper Leg	167.55 ± 47.57	162.26
Right Upper Leg	166.36 ± 49.73	169.86

The large angular MAE values correlate to the large bone length MAE values seen between marker-based and marker-less motion data. The left and right upper leg bone lengths performed significantly worse with a MAE of 167.55 ± 47.57 mm and 166.36 ± 49.73 mm, accounting for an average reduction of 28.62 percent and 28.43 percent in bone lengths compared to the marker bone lengths, respectively. This can be attributed to the large difference in hip joint locations, where marker-less tracking can perform significantly worse under body occlusions as seen across the angular MAE values across seated exercises in Fig. 14. The left and right forearm and lower leg joints performed significantly better in the bone length MAE comparison. The low to no occlusions around these joint areas are key to this result. Finally, the left and right upper arm is shown to perform slightly worse in comparison which can be a result of the small differences in the shoulder joint locations between both marker-based and marker-less pose layouts.

In summary, the marker-less motion data captured by the Nuitrack system is shown to perform worse in comparison to the marker-based ground truth. Body occlusions play a large role in reducing marker-less tracking accuracy. The variances in angular and bone length MAE values can potentially cause similar motions to produce dissimilar movement patterns across all joint data. Such errors were expected due to the single perspective of the marker-less tracking setup. Despite this significant drop in performance when using marker-less tracking, there are still similarities in the movement pattern collected by both systems that enable comparisons. The errors also replicate the reality of current state of the art marker-less tracking, especially how they may behave in the real world under complex movement scenarios.

## Usage Notes

### Visualisation tools

**Fig. 15:**
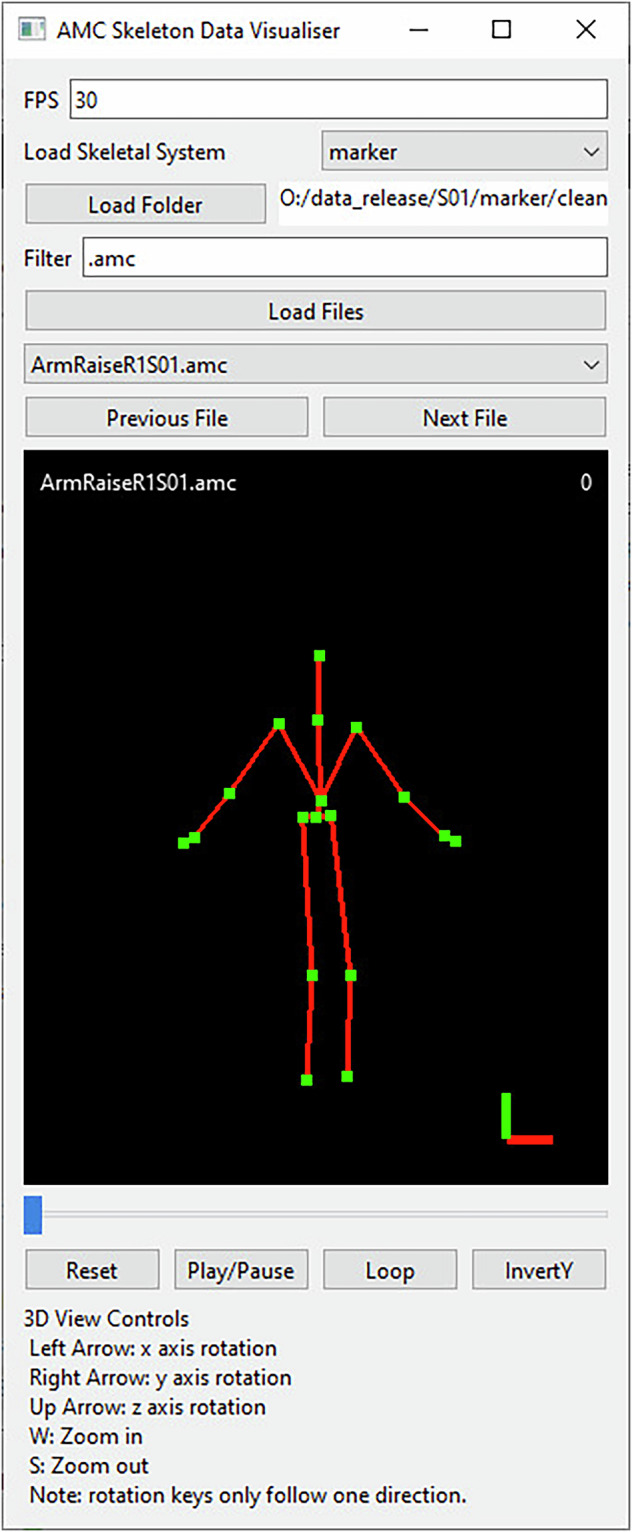
AMC human motion data 3D visualiser. The Frames Per Second (FPS) and marker pose layout can be preconfigured using the interface. The user can select the data using the load folder button and filtering box before loading the files using the “Load Files” button. Files are filtered using simple string matching. The drop-down box will populate with all loaded motions which are loaded when selected or via the file navigation button. Inside the skeletal viewport (denoted by the black background), the top left label defines the currently loaded filename and the top right defines the current frame number. The bottom right corner visualises the 3D axes, green is for the y axis, red for the x axis, and blue for the z axis. The slider below this viewport acts similar to a video playback slider for skimming through the recording. Playback can be controlled via the onscreen buttons and the viewport can be altered using the keyboard shortcuts listed on the interface.

A custom data visualisation interface is provided for interactive viewing of all AMC motion data, shown in Fig. 15. Instructions on using this visualiser is provided in the README file located at the root of the dataset folders.

## Data Availability

All Python pre-processing and technical validation code is provided together with the dataset. Python version 3.8.2 was used for all scripts and all Python dependencies, and their versions, are provided in the requirements text file contained within the code folder in the published dataset^16^. A custom ASF/AMC file parser is available in the Python programming language and can be installed using the pip package manager^18^.
